# Correlation between rs13347 polymorphism of CD44 gene and the risk of occurring breast cancer: A protocol for systematic review and meta-analysis

**DOI:** 10.1097/MD.0000000000025889

**Published:** 2021-06-04

**Authors:** Zilong Shao, Zhibin Wang, Liwei Shao, Xiang Jin

**Affiliations:** a Department of Emergency Medicine, Taikang Tongji (Wuhan) Hospital; b Department of Oncology, the Fifth Hospital of Wuhan, Wuhan, Hubei province, China.

**Keywords:** breast cancer, cluster of differentiation 44, gene polymorphism, meta-analysis, protocol

## Abstract

**Background:**

With the attention paid to the heritability of breast cancer, the search for the genetic susceptibility gene of breast cancer has become a hot spot. At present, a number of studies have explored the relationship between rs13347 polymorphism of cluster of differentiation 44 (CD44) gene and breast cancer, but the research conclusions are inconsistent. Therefore, we will use this meta-analysis to explore the role of this gene polymorphism in breast cancer susceptibility.

**Methods:**

The search time is set from the establishment of the database in March 2021 in this study. The search database include China National Knowledge Infrastructure, Wanfang, VIP Information Chinese Journal Service Platform, and China Biology Medicine disc, PubMed, EMBASE, Web of Science and the Cochrane Library. The subjects are observational studies on the relationship between rs13347 polymorphism of CD44 gene and breast cancer (including case-control study, cross-sectional study and a cohort study). The language is limited to English and Chinese. The data of the included study are extracted and the literature quality is evaluated by two researchers independently. The data are statistically analyzed by Stata 16.0 software.

**Results:**

Based on the existing studies, this study will systematically evaluate the relationship between CD44 gene rs13347 polymorphism and breast cancer.

**Conclusion:**

This study will provide evidence-based medical evidence to clarify the role of CD44 gene polymorphism in breast cancer, and provide help for the early detection and preventive intervention of breast cancer.

**Ethics and dissemination:**

Private information from individuals will not be published. This systematic review also does not involve endangering participant rights. Ethical approval will not be required. The results may be published in a peer-reviewed journal or disseminated at relevant conferences.

**OSF Registration number:**

DOI 10.17605/OSF.IO/WBC7F

## 1 Introduction

Breast cancer is a common malignant tumor in women. According to the statistics released by the World Health Organization in 2018, breast cancer is the first malignant tumor that threatens the health of women in the world. The new cases of breast cancer in the world are more than 2 million. In 2018, the incidence and mortality of female breast cancer are 46.3 /105 and 13.0 /105 respectively, and are on the rise.^[1]^ Each year, the number of new breast cancer cases and deaths in China account for about 12.2% and 9.6% of the world's breast cancer deaths, respectively.^[2]^ Due to the delay in diagnosis, the number of patients with advanced breast cancer has increased. Previous studies have shown that early diagnosis of breast cancer can significantly improve the survival rate of patients,^[3]^ but the current clinical lack of effective means of early screening. Studies have confirmed that breast cancer has genetic susceptibility.^[4]^ Exploring the relationship between genes and breast cancer will contribute to the early diagnosis and prevention of breast cancer.

Cluster of Differentiation 44 (CD44) is a multistructural and multifunctional transmembrane glycoprotein, which can be used as a receptor for hyaluronic acid and many other extracellular matrix components, as well as for a variety of growth factors and cytokines.^[5]^ Many studies have shown that CD44 is involved in many key processes of cells, including cell growth, reproduction, differentiation, adhesion and migration.^[6,7]^ CD44 is also a common tumor marker, which is closely related to the proliferation, migration and invasion of tumor cells, and participates in tumor-related angiogenesis. Studies have found that CD44 is widely expressed in a variety of cancer cells, affecting the prognosis of the disease, and the specific knockdown of CD44 will inhibit the occurrence and development of cancer.^[8]^

The association between CD44 gene rs13347 locus polymorphism and breast cancer has been paid attention by many scholars. Although there are many studies on the relationship between CD44 gene rs13347 locus polymorphism and breast cancer susceptibility,^[9–12]^ the conclusions are still controversial. Some studies have found that the polymorphism of the rs13347 site of the CD44 gene increases the risk of breast cancer,^[13]^ and some studies have come to the opposite conclusion that the polymorphism of the rs13347 site of the CD44 gene reduces the risk of breast cancer.^[14]^ In order to explore the source of this difference (such as whether it is related to race, nationality, regional population or differences in research methods, etc.), and to clarify the relationship between CD44 gene rs13347 polymorphism and breast cancer, we will adopt the method of meta-analysis to comprehensively analyze the existing data, in order to provide evidence-based medical evidence for the relationship between CD44 gene rs13347 locus polymorphism and breast cancer genetic susceptibility.

## 2 Methods

### 2.1 Protocol register

This protocol of systematic review and meta-analysis has been drafted under the guidance of the preferred reporting items for systematic reviews and meta-analysis protocols.^[15]^ Moreover, it has been registered on open science framework (OSF) (Registration number: DOI 10.17605/OSF.IO/WBC7F).

### 2.2 Ethics

Since the programme does not require the recruitment of patients and the collection of personal information, it does not require the approval of the Ethics Committee.

### 2.3 Eligibility criteria

(1) The subjects are those who are diagnosed as breast cancer by pathology and the control group (non-breast cancer patients). There are not any limitations on age, race and region of the patients.
(2) The type of study is an observational study to study the relationship between CD44 gene rs13347 locus polymorphism and breast cancer (including case-control study, cross-sectional study, cohort study). The language is limited to Chinese and English.
(3) The distribution frequency data of alleles or genotypes are available.
(4) The distribution frequency of genotypes conforms to the Hardy-Weinberg law.

### 2.4 Exclusion criteria.

(1) Repeatedly published research.
(2) Articles in which the published literature is an abstract or review, the data of the article is incomplete or incorrect, and the complete data can not be obtained after contacting the author.
(3) The study on the failure to provide detailed data on the frequency of genotypes.
(4) Animal research or experimental research.

### 2.5 Retrieval strategy

Take “CD44”, “gene polymorphism” and “breast cancer” as Chinese search words, and search them in Chinese database, including China National Knowledge Infrastructure, Wanfang, VIP Information Chinese Journal Service Platform, Chinese BioMedicine Literature Database. “Cluster of Differentiation 44,” “CD44,” “polymorphism,” “breast cancer,” “breast tumor,” and so on are used as English search words in the English database, including PubMed, EMBASE, Web of Science and the Cochrane Library. From the establishment of the database in March 2021, all the literature about the relationship between CD44 gene rs13347 polymorphism and breast cancer is collected. Take PubMed as an example, the retrieval strategy is shown in Table 1.

**Table 1:** Search strategy in PubMed database.

Number	Search terms
#1	Cluster of Differentiation 44 [Title/Abstract]
#2	CD44 [Title/Abstract]
#3	rs13347 [Title/Abstract]
#4	#1 OR #2 OR #3
#5	polymorphism [Title/Abstract]
#6	variant [Title/Abstract]
#7	#5 OR #6
#8	Breast cancer [MeSH]
#9	Breast Neoplasm [Title/Abstract]
#10	Breast Tumor [Title/Abstract]
#11	Mammary Cancer [Title/Abstract]
#12	Malignant Neoplasm of Breast [Title/Abstract]
#13	Malignant Tumor of Breast [Title/Abstract]
#14	Cancer of Breast [Title/Abstract]
#15	Mammary Carcinoma, Human [Title/Abstract]
#16	Human Mammary Neoplasm [Title/Abstract]
#17	Breast Carcinoma [Title/Abstract]
#18	#8 OR #9 OR #10 OR #11 OR #12 OR #13 OR #14 OR #15 OR #16 OR #17
#19	#4 AND #7 AND #18

### 2.6 Data screening and extraction

**Figure 1:**
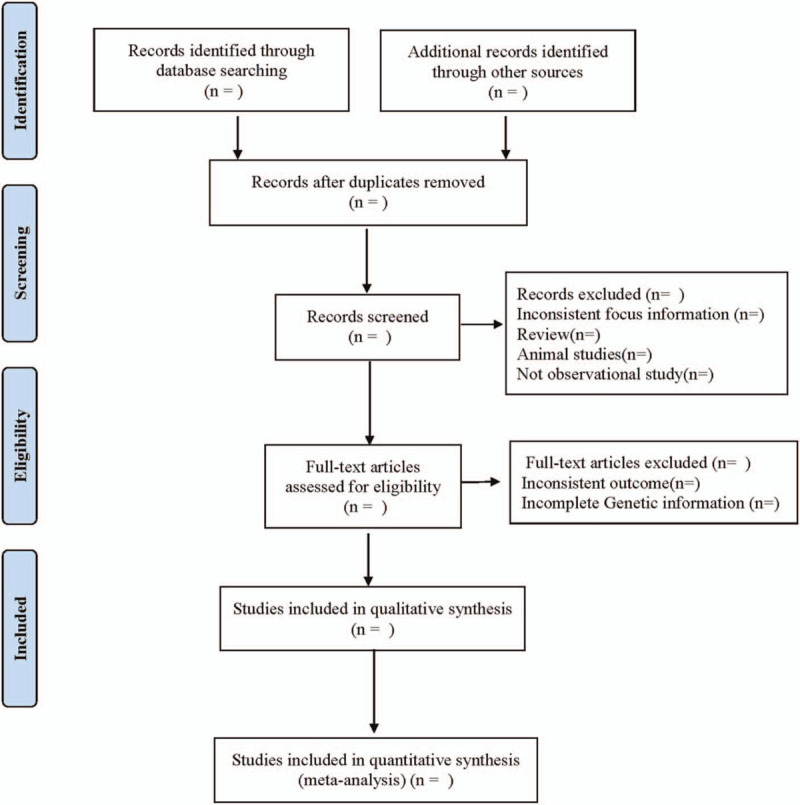
Flow diagram.

Two researchers independently complete the literature screening, exclude the studies that obviously do not meet the inclusion criteria, and further read the abstract and the full text to determine whether they meet the inclusion criteria. The data included in the literature are extracted and cross-checked. In case of disagreement, consult with the third researcher and reach a consensus. The extracted data include: the first author, the number of years of publication, the country of publication, the race of the research population, the basic characteristics of the study population (including age, sex, disease, etc.), the distribution of each gene phenotype (whether obeying the law of Hardy-Weinberg equilibrium), the detection method of gene polymorphism and so on. The literature screening process is shown in Figure 1.

### 2.7 Literature quality assessment

The case-control study and cohort study are evaluated by Newcastle-Ottawa Scale,^[16]^ including 3 columns and 8 items with a total of 9 points, and the evaluation criteria is ≥ 6 for high quality, while the cross-sectional study is evaluated with 11 standard items recommended by Agency for Healthcare Research and Quality for evaluation of cross-sectional study. 0 to 3 as low quality, 4 *to* 7 as medium quality, 8 to 11 as high quality.^[17]^

### 2.8 Statistical analysis

#### 2.8.1 Data analysis and processing

Before evaluating the association between gene polymorphism and breast cancer, *x*^2^ test is first used to test whether the genotypic distribution of the control group in each study is in line with Hardy- Weinberg genetic equilibrium (*P ≥* .05). Meta-analysis is carried out by using STATA.16 software, the continuous variables are expressed in the form of standard mean difference and 95% confidence interval, and the binary classification variables are expressed as odds ratio and 95% confidence interval. Heterogeneity is analyzed by *Q* test and *I*^2^, and the heterogeneity is evaluated according to the *I*^2^. If *P* *>* .1*, I*^2^ *<* 50%, the heterogeneity among the included studies is small, so the fixed effect model is used for analysis; if *P* *<* .1*, I*^*2*^*≥* 50%, the heterogeneity between the included studies is obvious, and the s urces of heterogeneity are analyzed. Random effect model is used for analysis.

#### 2.8.2 Dealing with missing data

If the data of the required study are incomplete or not reported in the study, the researcher will contact the first author or other author of the study by phone or email. If the required data are not available, we will use descriptive analysis instead of meta-analysis and exclude these studies if necessary.

#### 2.8.3 Subgroup analysis

In order to deal with the heterogeneity between different studies, subgroup analysis will be conducted according to the population (such as age, menopause, etc.), race (yellow, Caucasian, etc.) and regional (European, Asian, etc.).

#### 2.8.4 Sensitivity analysis

In order to test the stability of the meta-analysis results, we will use the one-by-one exclusion method to analyze the sensitivity of the results through Stata 16.0.

#### 2.8.5 Assessment of reporting biases

We will use to funnel chart to qualitatively identify publication bias, and use Egger and Begg test to quantitatively evaluate publication bias. If the funnel diagram is asymmetrical and *P* < . 05, it considers to have obvious publication bias.

#### 2.8.6 Evidence quality evaluation

We will use the Grading of Recommendation Assessment, Development and Evaluation scoring method to grade the outcome indicators of evidence.^[18]^ The quality of all evidence will be assessed on four levels (high, medium, low or very low) in terms of five aspects (bias risk, indirectness, inconsistency, inaccuracy, publication bias).

## 3 Discussion

The incidence of breast cancer is gradually increasing worldwide. According to the World Health Organization, there will be more than 19 million confirmed cases of breast cancers in the world by 2025. The pathogenesis of breast cancer is complex, and genetic susceptibility factors are closely related to the pathogenesis of breast cancer. In recent years, research and discussion of breast cancer susceptibility genes have become the focus of scholars all over the world.^[19,20]^

At present, there have been many studies on the relationship between single-nucleotide polymorPhisms and susceptibility to breast cancer. However, these SNP genes, including BRCA1 and BRCA2, can only explain about 25% of the susceptibility and incidence of breast cancer.^[9]^ Previous studies have shown that CD44 gene SNP, can be detected in 84% of breast cancer patients. Therefore, CD44 gene may be one of the high-penetrance genes that affect breast cancer susceptibility.^[21]^ The human CD44 gene is located on the short arm of chromosome 11 (11p13) and consists of 20 highly conserved exon and introns of different lengths.^[22]^ More and more studies have focused on the impact of CD44 gene ssingle-nucleotide polymorphisms on cancer risk. Among them, rs13347 polymorphism located in the 3’- untranslated Region of CD44 gene is the most studied.^[13]^ It affects the expression of CD44 gene by affecting the regulation of CD44 gene by hsa-miR-509–3P, and then affects the occurrence and development of breast cancer.^[23]^

Due to the differences in analytical methods, race and region of gene polymorphism, the relationship between CD44 gene rs13347 polymorphism and breast cancer is controversial.^[23]^ Therefore, this study will explore the relationship between CD44 gene rs13347 polymorphism and breast cancer by systematic review and meta-analysis, and provide evidence-based basis for CD44 gene polymorphism in risk assessment, early detection and prevention of breast cancer.

However, this study has some limitations: due to the limitations of language retrieval, we will only include Chinese and English literature in this study, and may ignore the research in other languages. Different race, skin color and disease and other factors may cause a certain degree of clinical heterogeneity.

## Author contributions

**Data collection**: Zilong Shao and Zhibin Wang

**Funding support**: Xiang Jin

**Resources**: Zhibin Wang and Liwei Shao

**Supervision**: Zilong Shao

**Software operating**: Zhibin Wang and Liwei Shao

**Writing – original draft**: Zilong Shao and Zhibin Wang

**Writing – review & editing**: Zilong Shao and Xiang Jin
